# Supplementary material for: Fluctuating Asymmetry in the Polymorphic Sand Cricket (Gryllus firmus): Are More Functionally Important Structures Always More Symmetric?
Source: Insects. 2022 Jul 16;13(7):640. doi: 10.3390/insects13070640 (PMC9319220; doi:10.3390/insects13070640)
Supplement: Supplementary file 1 [file insects-13-00640-s001.zip › Figure S1.pdf]

Supplementary Figure S1

A

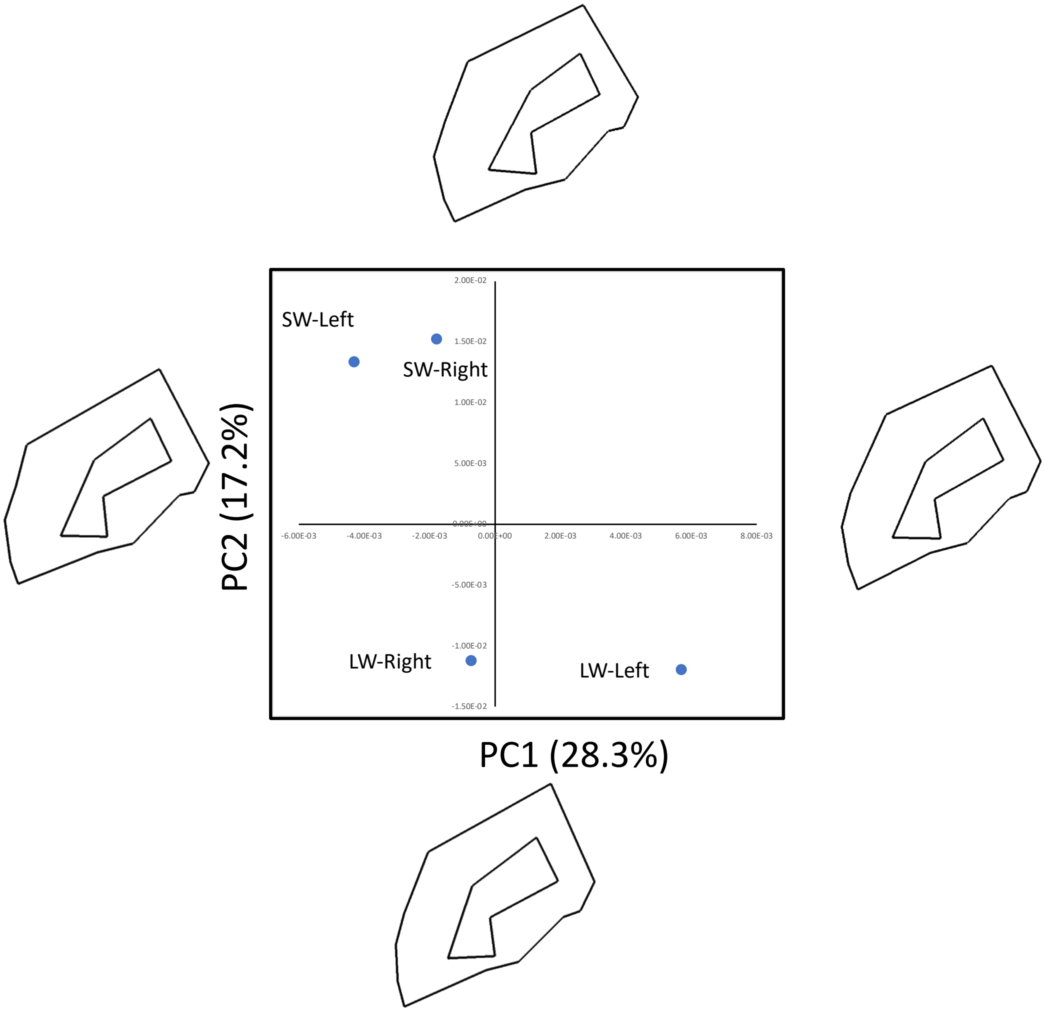

B

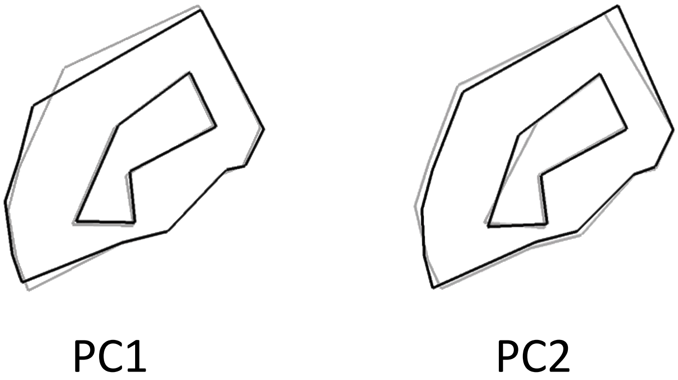

Supplementary Figure S1

Supplementary Figure S1. A comparison of the left and right forewings in LW and SW male crickets depicting shape differences associated with the observed directional asymmetry. The wing margin is broadly depicted by the outer polygon and is a connection of the GMM landmark points 1, 2, 3, 4, 5, 6, 17, 16, 15, 14, and 13 (as detailed in Figure 2). The wing structures corresponding to sound production are depicted as the inner polygon and is a connection of the points 7, 8, 10, 11, 12, and 9. A) A plot of the average PC scores for the left and right wings of the two male morphs. PC1 displays differences between sides within morphs, and PC2 displays differences between the two morphs. B) An overlay of the limits of the PC axes. Black lines illustrate negative values (i.e. left and down) and gray lines illustrate positive values (i.e. right and up).
